# Prognostic Value of Serum Albumin in Elderly Cardiovascular Disease Management

**DOI:** 10.14740/cr2286

**Published:** 2026-10-24

**Authors:** Yi Ying Liu, Hao Qing Zheng, Fan Lu Guan, Si Si Han, Shi Wei Huang, Fan Hao Ye, Wen Bing Jiang

**Affiliations:** a Department of Cardiology, The Third Clinical College, Wenzhou Medical University, Affiliated Wenzhou People’s Hospital, Hangzhou Medical College, Wenzhou, China; b These authors contributed equally to this study.

**Keywords:** Serum albumin, Prognosis, Cardiovascular disease, Hypoalbuminemia, Elderly

## Abstract

Cardiovascular disease (CVD) is the leading cause of morbidity and mortality worldwide and represents a major contributor to disability and poor health among elderly individuals, particularly those aged ≥ 65 years. Age-related physiological changes, endothelial dysfunction, inflammation, and oxidative stress play critical roles in the development and progression of CVD. Identifying reliable prognostic biomarkers is therefore essential to improve risk stratification and clinical management in this vulnerable population. Serum albumin (SA), the most abundant plasma protein, has emerged as a potential prognostic marker in elderly patients with CVD. Accumulating evidence from observational studies indicates that hypoalbuminemia is associated with increased all-cause mortality across a wide spectrum of CVDs, including coronary artery disease, heart failure, hypertension, atrial fibrillation, stroke, peripheral artery disease, and in patients undergoing various cardiovascular interventions. Importantly, low SA levels have been shown to remain an independent prognostic marker of adverse outcomes in observational studies after adjustment for confounding factors, such as malnutrition, inflammation, and comorbid conditions, although these associations may partly reflect underlying disease burden and other clinical factors. Proposed mechanisms linking hypoalbuminemia to poor outcomes include impaired antioxidant capacity, increased inflammation, endothelial dysfunction, and altered fluid balance. This review also highlights substantial heterogeneity in SA threshold values reported across studies, which may limit its standardized application in clinical practice. Although albumin supplementation has been suggested as a potential therapeutic strategy, current evidence regarding its clinical benefit remains inconclusive. SA may provide additional prognostic information in elderly patients with CVD and may support improved risk stratification across diverse clinical settings. At present, management strategies should prioritize identifying and addressing the underlying causes of hypoalbuminemia rather than routine albumin supplementation. Further well-designed studies are needed to establish standardized SA cut-off values and to clarify its role in guiding clinical decision-making in elderly cardiovascular populations.

## Introduction

Cardiovascular diseases (CVDs) comprise a group of disorders associated with the heart and its blood vessels, with atherosclerosis being the primary underlying cause [1]. It is the leading cause of morbidity and mortality worldwide and represents a significant public health challenge [2]. Age is an independent risk factor that decreases the ability and efficiency of the cardiovascular (CV) system [3]. CVD is the primary health burden among middle-aged and older adults [4], imposing substantial strain on patients and healthcare systems, with prevalence increasing rapidly because of global population ageing [5]. According to the American Heart Association, the prevalence of CVD increases from approximately 38% to 43% in adults aged 40 to 59 years to > 85% among those aged ≥ 80 years, underscoring the disproportionate burden in elderly populations [6]. Therefore, evaluating the burden of CVD in elderly patients, particularly those aged > 70 years, is essential to inform evidence-based strategies for disease reduction. Notably, about 330 million individuals in China are affected by CVDs. Furthermore, this number is projected to increase in the population aged ≥ 65 years from 190 million to 240 million by 2030, with 70% of individuals aged > 60 years affected by CVDs [7].

In 1986, the 18th Bethesda Conference of the American College of Cardiology explicitly focused on CVDs in elderly patients, aiming to address the growing population of older adults in the United States and the need for specialized geriatric cardiology services for the care of elderly patients with CVDs. In 2020, > 13 million individuals were aged ≥ 80 years, and by 2040, 22% of the population is expected to be aged ≥ 65 years [8]. A wide range of prognostic factors contribute to increased mortality risk in elderly patients, both in hospital settings and in the community. Prognostic markers guide clinicians about expected outcomes and support end-of-life care planning. Identifying markers associated with high mortality or poor outcomes enables healthcare professionals to come up with potential treatment options and be involved in discussions with elderly patients and their families [9]. The etiology of CVD is considered to be multifactorial and cannot be described by a single factor. However, low serum albumin (SA) level remains a prognostic indicator in patients with CVD [10–12]. Although several circulating biomarkers, such as cardiac troponins, B-type natriuretic peptide (BNP), and C-reactive protein (CRP) and other inflammatory markers including white blood cell count, neutrophil count, and erythrocyte sedimentation rate (ESR), are well characterized for diagnosing myocardial injury, assessing heart failure (HF) severity, and evaluating systemic inflammation, their prognostic utility in older adults is often influenced by acute disease states and may not fully capture age-related vulnerability [13]. In contrast, SA represents an integrative prognostic biomarker that reflects nutritional status, the cumulative effects of chronic inflammation, frailty, and physiological reserve, the key geriatric factors influencing CV outcomes in older adults [11]. During inflammatory states, pro-inflammatory cytokines, including interleukin-6 (IL-6) and tumor necrosis factor-α (TNF- α), suppress hepatic albumin synthesis, leading to reduced circulating albumin concentrations. [14, 15] Low SA level mirrors the cumulative burden of age-related processes, including sarcopenia, malnutrition, and multisystem dysfunction, thereby linking geriatric biology with CV pathophysiology [9]. This multifaceted role distinguishes SA from conventional cardiac biomarkers and supports its relevance as a prognostic indicator in elderly patients with CVD [16, 17].

This narrative review evaluates the prognostic significance of SA in the development and progression of CVD among elderly patients, as well as the impact of age on the development and incidence of CVD. This narrative review was based on a non-systematic literature search of PubMed/MEDLINE, Google Scholar, and relevant reference lists. Search terms included combinations of “serum albumin,” “hypoalbuminemia,” “elderly,” “older adults,” “cardiovascular disease,” “coronary artery disease,” “heart failure,” “atrial fibrillation,” “stroke,” “acute coronary syndrome,” “prognosis,” “mortality,” “inflammation,” and “nutrition.” Articles were selected based on their relevance to the prognostic significance of SA in elderly patients with CVD, including clinical studies, reviews, meta-analyses, and mechanistic literature. Unlike previous studies, the present review highlights the emerging evidence on the association between clinical thresholds of SA identified across studies. Furthermore, the narrative review explores the underlying mechanisms associated with low SA levels and adverse CV outcomes, which will help clinicians in risk stratification among elderly populations, thereby enabling the development of optimized therapeutic strategies.

## Molecular Structure and Physiological Characteristics of SA

### Structure and function of SA

Albumin belongs to the albumin superfamily, which also includes the transport proteins α-fetoprotein, vitamin D-binding protein (Gc-globulin), and afamin (α-albumin). It is an aglycosylated, negatively charged protein composed of 585 amino acids, with a molecular weight of 66.5 kDa [18]. Within the SA molecule, the polypeptide chain is linked by 17 disulfide bonds and is organized into three homologous domains (1–3), which assemble to form a heart-shaped molecule [15, 19]. This arrangement in SA provides flexibility to the structure of the protein and allows it to adapt to a variety of endogenous and exogenous ligands [20], thereby making it one of the best examples of structure–function correlation in monomeric proteins [21]. Albumin is also a major binding protein for several drugs, particularly highly protein-bound medications. Therefore, reduced SA levels may increase the unbound/free fraction of these drugs [21]. In elderly patients with CVD, who frequently receive multiple concomitant medications, hypoalbuminemia may alter drug exposure and increase susceptibility to adverse drug reactions, which may contribute to poor clinical outcomes [15].

Consequently, albumin demonstrates multifunctional properties, including anti-inflammatory, antioxidant, anticoagulant, toxin-binding, and antiplatelet aggregation effects. These properties may contribute to vascular homeostasis and are involved in mechanisms associated with the development and progression of CVD [22, 23]. In addition, albumin plays a critical role in CV physiology and pathophysiology, particularly through its influence on cardiac dynamics. Its properties overlap with several key mechanisms involved in the progression of CVD [24, 25]. Based on these properties, low SA level has been increasingly recognized as a predictive biomarker for both the development and prognosis of CVD.

### Regulation of SA levels

SA is synthesized by hepatocytes as preproalbumin, which matures into albumin within the endoplasmic reticulum and the Golgi apparatus. The normal SA levels typically range from 3.5 to 5.0 g/dL, although clinical thresholds for disease states vary by analytical method [26]. In healthy adults, the total albumin content is 250 to 300 g, and its concentration depends on synthesis, degradation, and distribution across intravascular and extravascular compartments [20].

Reduction in SA levels is common in disease states and occurs primarily because of the loss of albumin from the gastrointestinal (GI) tract. However, compensatory mechanisms help maintain normal albumin levels. To maintain homeostasis, the body mobilizes protein reserves from muscle and other tissues to support hepatic synthesis, whereas redistribution between compartments and reduced catabolism further stabilize circulating albumin. These adaptive responses help maintain SA levels until prolonged or severe malnutrition overwhelms the homeostatic system [27].

Albumin regulates the oncotic (colloid osmotic) pressure of blood, thereby influencing the physiological processes within the circulatory system [10]. During acute inflammation, SA functions as a scavenger of reactive oxygen species (ROS), and a reduction in SA impairs plasma antioxidant capacity, potentially promoting CV events. Consequently, albumin levels are inversely correlated with CV risk and mortality in both the general population and patients with established CVDs [28, 29]

## Hypoalbuminemia in Elderly Populations (Aged ≥ 60 Years)

Hypoalbuminemia has been reported as a prognostic marker for CVDs and associated outcomes [30]. Hypoalbuminemia may serve as a potential modifiable risk factor in the development and progression of CVDs, primarily mediated through oxidative stress, increased inflammation, and platelet aggregation [30].

Hypoalbuminemia is highly prevalent among older adults [31], with the rates ranging from 4% to 50%, depending on the care setting (e.g., home-based vs hospice or bedridden populations). In elderly populations, SA levels decline with age, with approximately 20% reduction reported in individuals aged > 70 years. This decline impairs the physiological responses to injury, leading to prolonged hospitalizations and increased morbidity [32]. For example, a large retrospective study conducted involving hospitalized patients (n = 9,428; mean age, 65 years) reported a decrease in SA levels with increasing age, with patients aged > 65 years demonstrating albumin levels < 3.5 g/dL [33]. Moreover, in a recent study involving elderly patients (n = 1,058; mean age, ≥ 60 years) with nonischemic dilated cardiomyopathy (NIDCM), the SA level was identified as a significant prognostic factor associated with clinical outcomes. Furthermore, hypoalbuminemia was found to be associated with an increased risk of in-hospital and long-term mortality in elderly patients (odds ratio (OR), 4.334; 95% confidence interval (CI), 2.18–8.59) [34]. A meta-analysis (up to March 2019) of 10 studies involving 16,763 patients reported that hypoalbuminemia in acute HF (risk ratio (RR), 4.90) was associated with an increase in in-hospital mortality (RR, 1.75) and long-term all-cause mortality. In patients with chronic HF, hypoalbuminemia was associated with a 3.5-fold higher risk of long-term mortality [35]. In another multicenter registry of the Tokyo Cardiovascular Care Unit Network conducted between January 2017 and December 2020, patients with takotsubo syndrome (n = 631) had a median age of 78 years. Hypoalbuminemia (< 3.5 g/dL) was reported in 32% of patients, with older patients reporting low SA levels. Patients with hypoalbuminemia demonstrated significantly higher in hospital all-cause mortality (9.5% vs 1.9%) compared with those with normal albumin levels [36]. However, these findings should be interpreted cautiously, as the retrospective design does not establish causality, and the observed association may be influenced by underlying inflammation, comorbidities, and other clinical factors contributing to hypoalbuminemia. Another study investigated the importance of hypoalbuminemia in frail elderly patients (n = 67) with a mean age of approximately 86 years. The study further demonstrated that among various predictors, SA (after clinical stabilization) was the only independent predictor of hospital mortality (P < 0.01), even after adjustment for malnutrition, inflammation, and liver dysfunction [37]. However, these findings should be interpreted cautiously considering the observational design, small sample size, single-center setting, and the possibility of overestimation of the association due to limited events. Therefore, the findings from different studies suggest that advancing age is associated with lower albumin levels and an increased risk of hypoalbuminemia [38]. Although age is not the only factor contributing to decreased SA levels in the elderly populations, it may reflect other age-related factors such as decreased physical activity, reduced appetite, changes in body composition, and lower energy requirements [38]. These age-related factors make hypoalbuminemia a common yet critically significant issue in elderly patients with CVDs.

### Causes of hypoalbuminemia in elderly patients with CVDs

#### Inadequate nutrient intake

Inadequate nutrient intake and poor dietary habits are major concerns in elderly populations [39]. Malnutrition reduces albumin levels by reducing its rate of synthesis [14]. Consequently, hypoalbuminemia results from the combined effects of inflammation and insufficient protein and caloric intake in patients suffering from chronic diseases. Malnutrition is prevalent among hospitalized elderly patients and is associated with increased morbidity and mortality [40]. Malnutrition, as defined by the Global Leadership Initiative on Malnutrition criteria, has been shown to be strongly associated with mortality in both hospitalized and community-dwelling elderly populations, highlighting the vital role of nutritional status [9]. Furthermore, supporting these findings, Bretscher et al (2022) conducted a secondary analysis of the EFFORT trial involving 2,028 patients (mean age, 73.1 years) with nutritional deficiency and reported that 48.7% of patients had hypoalbuminemia, defined using a cutoff value of 3.0 g/dL [40]. Evidence from recent studies highlights the importance of early identification of the nutritional status of patients and timely intervention to address malnutrition in nutrient-deficient populations. In elderly populations, the metabolic process primarily involves catabolism, increasing the demand for more proteins to compensate for tissue protein consumption. Therefore, reductions in SA levels may reflect a potential inflammatory response associated with CVD [7]. Investigating and addressing nutritional deficiencies is therefore important to improve clinical outcomes and quality of life, particularly in high-risk elderly populations.

#### Other causes

Age-related variations in SA levels have been reported in several studies. An important contributing factor is chronic low-grade inflammation, which is frequently observed during aging and may lead to impaired albumin synthesis and upregulated albumin catabolism. This inflammatory state, characterized by elevated concentrations of IL-6 and TNF-α, represents a key inflammatory mechanism contributing to hypoalbuminemia [41]. Several age-related conditions, including metabolic syndrome, have been associated with reduced SA levels, particularly in the presence of abdominal obesity, hypertriglyceridemia, and hyperglycemia. Lower SA levels in these patients have been associated with increased mortality rates. Overall, age-related changes in SA levels may be influenced by altered metabolism, inflammation, nutritional status, and several other comorbid conditions [9]. Moreover, low SA levels have been associated with an increased risk of frailty in elderly patients. Consequently, SA serves as an effective prognostic marker for assessing the overall health and functional status in elderly populations [9].

## SA as a Prognostic Marker in CVDs

### Occurrence in specific CVD conditions

Although SA is an important prognostic biomarker in elderly patients with CVD, it represents only one component of nutritional and inflammatory assessment. Composite immunonutritional indices, such as the CONUT score (SA, total cholesterol, and lymphocyte count), the prognostic nutritional index (PNI), (SA and lymphocyte count), and the C-reactive protein–albumin–lymphocyte (CALLy) index (CRP, SA, and lymphocyte count) provide a broader evaluation of nutritional and immune status and may offer complementary prognostic information in CVD [42–44]. In particular, CALLy incorporates systemic inflammatory burden through CRP in addition to albumin and lymphocyte count. However, further studies are needed to determine their incremental value beyond SA alone. In routine clinical practice, there is no standard definition of low SA for investigating the adverse events related to CVDs. Several observational studies have reported an increased risk of CV events and mortality even at albumin levels ranging from 3.5 to 4.0 g/dL [25, 43, 44]. Albumin is assumed to have a significant relationship with cardiac dynamics and inflammatory regulation through its anti-inflammatory, antioxidant, anticoagulant, and ligand-binding properties, which influence endothelial function, oxidative stress, thrombosis, and atherosclerotic processes involved in the development and progression of CVDs [18, 22, 25]. However, these findings are primarily based on observational evidence, rather than randomized controlled trials, and the association between SA levels and CV outcomes should be interpreted cautiously, as causality and the clinical benefit of correcting hypoalbuminemia remain uncertain. Thus, various studies have been conducted to investigate the correlation between SA levels and different types of CVDs (Fig. 1, Table 1) [25, 34, 45–58].

**Figure 1:**
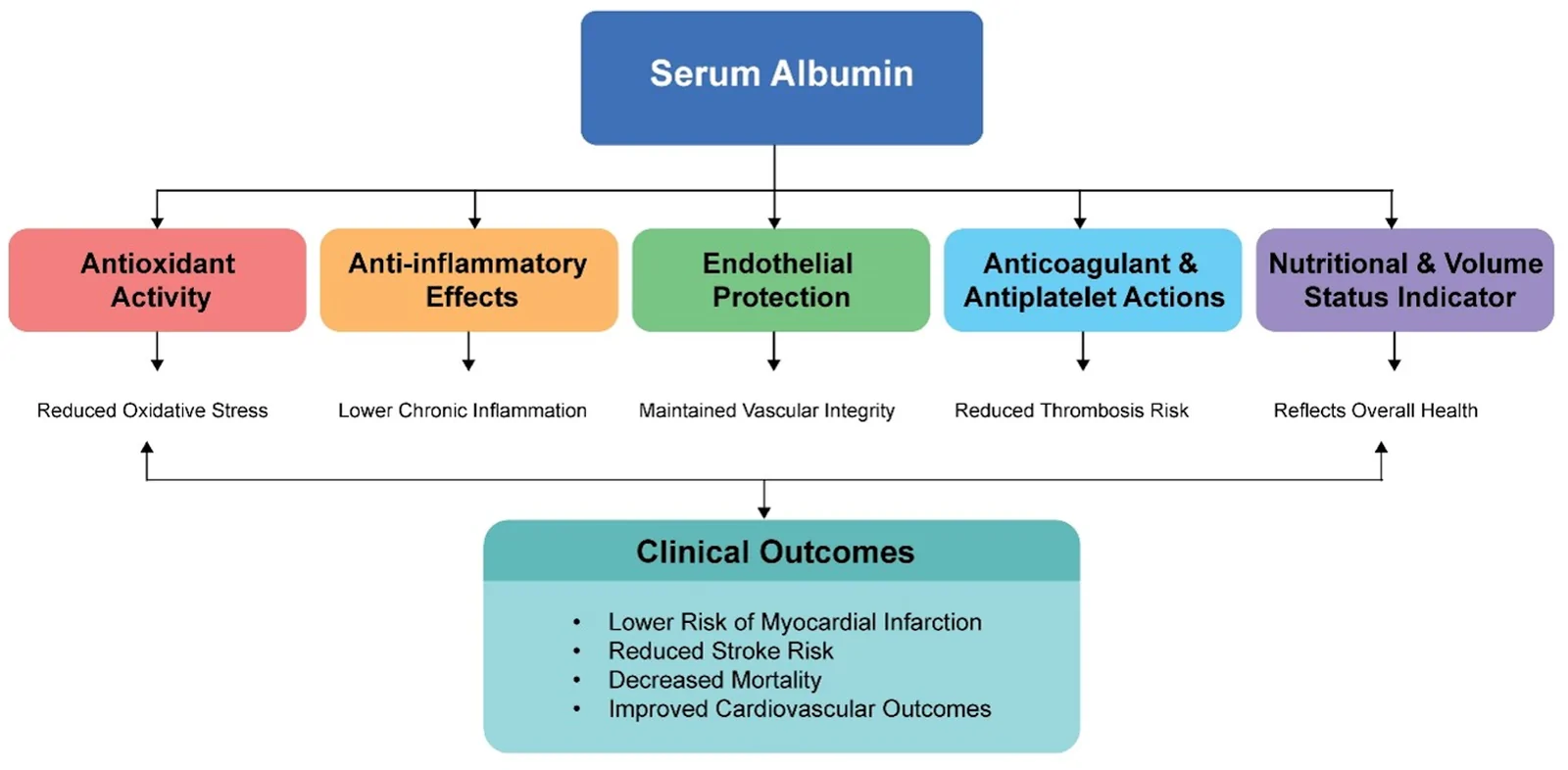
Proposed interaction between SA and CV outcomes (pathophysiological mechanisms by which SA leads to CVD). SA: serum albumin; CV: cardiovascular; CVD: cardiovascular disease.

**Table 1:** Summary of Studies Assessing the Prognostic Value of Serum Albumin Levels in Patients with CVD

Study	Population	CVD type	Albumin threshold	Key findings	References
Prospective (registry) cohort	N = 13,618; mean age, 62.2 ± 11.3 years; China; multicenter national registry; patients with AIS or TIA	AIS and TIA	< 3.5 g/dL	The relationship in each 10 g/L lower albumin with prognosis was consistently inverse (aOR, 1.17; aHR, 1.86)	Zhou et al, 2021 [48]
Cross-sectional study	N = 10,900; mean age, 63.9 ± 9.3 years; China; cross-sectional, hospital-based cohort; adult patients with hypertension	Chinese hypertensive patients	4.0 g/dL	Lower SA was associated with the prevalence of PAD only in males but not in females	Ding et al, 2020 [49]
Retrospective study	N = 378; mean age, 67.1 ± 11.2 years; Italy; single-center cohort; patients with chronic HF	HF	3.5 g/dL	Patients with chronic HF who exhibited low SA levels showed a higher risk of MACE, hHF, and total mortality	Armentaro et al, 2024 [50]
Randomized COAPT trial	N = 614; mean age, 71–74 years; multinational; randomized controlled trial; HF with SMR	HF and SMR	4.0 g/dL	559 patients (91.0%) had baseline SA levels (median, 4.0 g/dL; interquartile range (IQR) 3.7–4.2 g/dL). Patients with albumin levels < 4.0 g/dL were older and more likely to have ischemic cardiomyopathy and hospitalization within the year before enrolment compared with those with albumin levels ≥ 4.0 g/dL	Feng et al, 2023 [51]
Retrospective study	N = 3,057; median age, 58 years; Turkey; multicenter cohort; patients with ST-segment, elevation myocardial infarction undergoing PCI	ST-segment elevation myocardial infarction	-	sACR was inversely associated with short-term clinical outcomes in patients with STEMI after PCI	Turkyilmaz et al, 2022 [52]
Retrospective study	N = 1,058; mean age, 67–69 years; China; hospital-based cohort; elderly patients with NIDCM	NIDCM	3.5 g/dL	Hypoalbuminemia was associated with an increased risk of in-hospital and long-term mortality in elderly patients with NIDCM	Li et al, 2023 [34]
Retrospective cohort	N = 321; age ≥ 65 years; Taiwan; single-center cohort; elderly patients with stable CAD	Stable CAD	< 3.75 vs ≥ 3.75 g/dL	Patients with albumin ≥ 3.75 g/dL had significantly lower long-term cardiovascular mortality vs those with < 3.75 g/dL. HR about 0.20 for CV mortality in the ≥ 3.75 group after adjustment	Cheng et al, 2024 [45]
Retrospective cohort	N = 119; age ≥ 65 years; United States; single-center study; patients hospitalized with ADHF	ADHF	≤ 2.9 g/dL	Low albumin (≤ 2.9) at admission was associated with increased all-cause mortality after a follow-up of 11 years	Mene-Afejuku et al, 2019 [46]
Prospective, monocenter study	N = 64; institutionalized elderly; France; monocenter prospective study; patients hospitalized with acute HF	Acute HF (in-hospital outcomes)	<3.0 g/dL (median)	SA < 3.0 g/dL was a strong predictor of in-hospital mortality; high sensitivity (94%) and moderate specificity (68%) for in-hospital mortality prediction	Arques et al, 2008 [53]
Observational cohort	N = 873; mean age, 70.9 ± 7.5 years; China; cohort study; elderly patients with chronic aortic regurgitation	Valvular heart disease, chronic aortic regurgitation	3.5 g/dL	When albumin was < 3.5 g/dL, each 1 g/L decrease corresponded to 25% increased risk of all-cause mortality; hypoproteinemia (< 3.5 g/dL) had an HR of 2.93	Li et al, 2025 [54]
Epidemiologic cohort	820 elderly men (mean age, 64–84 years)	Coronary heart disease risk/incidence and mortality	Albumin was expressed as a continuous variable; moderate reductions (per SD decrease about 2.5 g/dL)	Moderate to low albumin levels predicted incident coronary heart disease, particularly in those with high cholesterol; lower albumin levels were associated with CHD mortality and all-cause mortality in elderly men	Weijenberg et al, 1997 [55]
Cohort study	546 patients (mean age 78 ± 9 years)	Acute nonischemic HF	< 3.57 g/dL	Hypoalbuminemia (< 3.4 g/dL) was independently predictive of hospital mortality; good sensitivity (78.8%) and specificity (75%)	Ancion et al, 2017 [47]
Cohort/retrospective study	759 patients (mean age, about 68 years)	AIS	≥ 4.9 g/dL (4.9 g/dL)	Patients with lower albumin levels had higher odds of poor functional outcome (mRS > 3/death) at the 3-month follow-up period	Dziedzic et al, 2004 [56]
Prospective study	11, 298 patients; (mean age = 62 years)	AIS	A/G ratio cutoffs were 1.1 and 1.9, depending on the outcome and time point; clinically relevant cutoff, 1.38	Lower serum A/G levels were associated with poor functional outcomes and all-cause mortality at 3-month and 1-year follow-up periods in patients with AIS	Wang et al, 2023[57]
Retrospective study	477 patients; (age: T1, 62 (56–69); T2, 67 (61–76); T3, 69 (60–75))	HF patients with CRT-D	> 0.038 g/dL	Low albumin levels were associated with long-term mortality and appropriate shock treatment in patients with HF, including those treated with CRT-D	Pay et al, 2024[58]

A/G ratio: albumin-to-globulin ratio; ADHF: acute decompensated heart failure; aHR: adjusted hazard ratio; AIS: acute ischemic stroke; aOR: adjusted odds ratio; CAD: coronary artery disease; CHF: congestive heart failure; CHD: coronary heart disease; CRT-D: cardiac resynchronization therapy defibrillator; CV: cardiovascular; CVD: cardiovascular disease; HF: heart failure; hHF: hospitalization for heart failure; MACE: major adverse cardiovascular events; mRS: modified Rankin scale; NIDCM: nonischemic dilated cardiomyopathy; PAD: peripheral artery disease; SA: serum albumin; sACR: serum albumin-to-creatinine ratio; SD: standard deviation; SMR: secondary mitral regurgitation; TIA: transient ischemic attack.

#### Coronary artery disease (CAD)

Several studies have reported an association between SA levels and clinical outcomes among patients with CAD, including those in the acute phase. Several underlying mechanisms, including endothelial dysfunction, inflammation, and oxidative stress, are associated with a high incidence of adverse events in patients with CAD. Owing to the physiological effects of SA, such as anti-inflammatory, antioxidant, and antiplatelet aggregation, hypoalbuminemia has been reported as a prognostic marker in patients with CAD [59]. Although the physiological functions of albumin are well reported, the optimal SA level in elderly patients with CAD remains unclear. Therefore, it is critical to explore the association between preprocedural SA levels for CV mortality among elderly patients with CAD. To better understand the potential prognostic significance of SA in CAD, several observational studies have reported results in both populations with stable and acute CAD [45, 60–62]. In one study, the association between SA levels and CV mortality was investigated in elderly patients (n = 321) with stable CAD. The study reported that patients with SA ≥ 3.75 g/dL had a lower frequency of CV mortality compared with those with SA < 3.75 g/dL. These findings suggested that SA levels may provide additional prognostic information in elderly patients with stable CAD [45]. In an epidemiological observational study, a correlation between SA levels and CAD was demonstrated. Lower SA levels were associated with older age, and hypoalbuminemia was also evident in elderly patients. In addition, 2,860 patients with CAD who underwent percutaneous coronary intervention (PCI) between 2000 and 2011 were included in this study. SA levels were significantly associated with the incidence of major adverse cardiovascular events (MACEs; hazard ratio (HR), 1.74 per 1 g/dL decrease; 95% CI, 1.34–2.26) and all-cause mortality (HR, 1.74; 95% CI, 1.30–2.33). Lower albumin levels before PCI were independently associated with higher rates of MACE and all-cause mortality for a median follow-up of 7.4 years [60]. Another study involving elderly patients (n = 204) with stable CAD treated with elective PCI (median age, 73 years) investigated SA level at admission. For a median follow-up of 783 days, it was found that a lower SA level was significantly (P < 0.001) associated with a higher incidence of MACE (n = 28), defined as all-cause death, nonfatal myocardial infarction (MI), and nonfatal stroke [61]. Furthermore, a study involving elderly patients (n = 288; mean age, ≥ 65 years) with acute myocardial infarction (AMI) investigated the changes in SA levels during hospitalization. S-shaped associations were reported between albumin change and mortality. For all-cause death, two inflection points (3.27 and 0.92 g/L) were reported. For cardiac death, two inflection points (3.19 and 1.17 g/dL) were reported [62]. Hypoalbuminemia and adverse CV outcomes were also observed in the Biosignature study, which prospectively enrolled patients with CAD in stable condition at baseline. Patients in the low SA group were older compared with those in the normal SA group. In addition, patients with hypoalbuminemia had an increased risk of MACE and all-cause mortality for 1.5 years (HR, 3.68; 95% CI, 1.03–13.19; all-cause mortality: HR, 6.81; 95% CI, 1.01–45.62) [18]. However, these findings should be interpreted cautiously, as the number of patients with hypoalbuminemia was limited, and the observed association may partly reflect underlying frailty, comorbidities, inflammation, and nutritional status rather than an independent effect of SA itself.

Overall, these findings indicate that increased SA levels are associated with better prognosis and survival among elderly patients with CAD; however, this association should be interpreted with caution, as the available evidence is primarily derived from observational studies. Additionally, some evidence suggests that combined assessment of elevated CRP together with low albumin may provide informative prognostic value than albumin alone, highlighting the importance of interpreting SA within a broader inflammatory and clinical context.

#### HF

HF affects more than 5 million individuals in the United States, and this number is expected to increase with rising life expectancy [46]. In elderly patients, SA acts as an emerging potential prognostic biomarker in HF. Albumin concentrations have been shown to be independently associated with 5-year risk in elderly patients with HF [63]. Hypoalbuminemia is frequently reported in patients with HF, especially among older adults, with a prevalence of about 25% [46, 64]. This prevalence is further highlighted by a retrospective study involving patients (n = 119; mean age, ≥ 65 years) with acute decompensated heart failure (ADHF), in which patients with SA ≤ 2.9 g/dL demonstrated significantly higher mortality over an 11-year follow-up period. Hypoalbuminemia was defined as SA ≤ 2.9 g/dL, and a significantly higher proportion of patients in the mortality group had admission SA levels of ≤ 2.9 g/dL. These results suggest that hypoalbuminemia may serve as an important marker of mortality in elderly patients with ADHF [46]. However, these findings should be interpreted cautiously, as the retrospective design does not establish a causal relationship, and heterogeneity among studies evaluating the association between SA levels and outcomes in HF may influence the interpretation of findings. These results suggest that hypoalbuminemia may serve as an important marker of mortality in elderly patients with ADHF. In another study, the correlation between albumin and hospital mortality was investigated involving 509 patients (mean age, 72 ± 12 years) admitted with acute HF. The SA level was shown to be an independent predictor of long-term mortality. Hypoalbuminemia (< 3.57 g/dL) was associated with an HR of 2.01 (95% CI, 1.24–3.25), whereas hemoglobin was associated with an HR of 2.6 (95% CI, 1.29–5.22) for predicting long-term mortality. In this study, the SA level measured during hospitalization, combined with anemia, served as a simple prognostic factor in acute HF for predicting long-term outcomes [65]. Another study investigated the prognostic significance of SA levels in hospital mortality among patients admitted with acute nonischemic HF (n = 546; mean age, 72–78 years). Mortality was reported in 36 patients (7%) during hospitalization. These patients were older and had significantly lower albumin concentrations (3.13 vs 3.69 g/dL). These results suggest that hypoalbuminemia is associated with an increased risk of hospital mortality, especially in elderly patients [47].

Overall, these findings indicate that low SA levels are associated with adverse events in elderly populations with HF; however, these associations should be interpreted cautiously. This highlights the importance of incorporating SA analysis into routine risk assessment to support better management strategies in high-risk elderly populations. However, current evidence does not support routine albumin administration as a therapeutic strategy in patients with HF. The reported clinical benefits appear limited or inconclusive, and a meta-analysis evaluating co-administration of albumin with furosemide in patients with hypoalbuminemia did not demonstrate clear clinical benefit. Therefore, albumin supplementation should be interpreted cautiously and should not be considered routine therapy for HF unless supported by specific clinical indications [66].

#### Atrial fibrillation (AF)

The association between SA and AF remains controversial [67]. SA, as a biomarker measured in clinical practice, plays an important role in the pathogenesis of AF; however, it has not been fully recognized in clinical practice [67]. The prevalence of AF increases with age, making it the most common arrhythmia in patients aged > 65 years. In patients aged > 80 years, the prevalence rate is about 10%, which contradicts with 70% prevalence in patients aged 65 to 85 years [68]. Studies with experimental clinical data have indicated that low SA influences the pathophysiology of AF by inducing inflammation and oxidative stress. A dose-response meta-analysis of nine studies involving 32,130 patients reported that lower albumin levels were associated with a higher risk of developing AF (OR, 0.62; 95% CI, 0.44–0.89). The mean age of patients ranged from 52 to 67 years. For each 10 g/L increase in SA level, the risk of AF decreased by 36% [69]. In an epidemiological cross-sectional study, 5,879 elderly Chinese patients (mean age, ≥ 65 years) were included. The results indicated that a lower albumin level (< 3.5 g/dL) was significantly associated with the occurrence of AF (P < 0.05), particularly among male patients [70].

Overall, these findings reflect the high incidence and significant impact of AF in elderly populations. They also highlight the requirement for improved screening, prevention, and management strategies tailored to this age group to investigate AF as a common and serious arrhythmia in this population.

#### Hypertension/stroke

Stroke is the most common type of CVD. Albumin plays an important role in determining the risk of stroke and exhibits anti-inflammatory, antioxidant, and antiplatelet coagulation. These actions might affect the development and progression of stroke [71]. The SA levels have been shown to be inversely proportional to the risk of stroke in individuals aged > 40 years, even after adjustment for confounding variables using multiple logistic regression [71]. To investigate this, the relationship between SA levels and the risk of stroke in adults (≥ 40 years) was investigated. A cross-sectional study was conducted from 2009 to 2018 by the National Health and Nutrition Examination Survey involving 17,303 participants. The results suggested that the SA levels were significantly and inversely associated with the risk of stroke, even after adjustment for confounding variables (OR, 0.02; 95% CI, 0.01–0.04; P < 0.0001). Subgroup analysis proposed that the inverse relationship between SA levels and the risk of stroke was statistically significant among male participants (< 60 years), individuals without diabetes, and those with hypertension. The SA levels and the risk of stroke were negatively correlated. Lower SA levels were associated with an increased risk of stroke [71]. In a recent meta-analysis, 9,979 patients with acute ischemic stroke were included (mean age, 78.3 (± 11.2) years). Patients with albumin levels < 3.7 g/dL demonstrated an approximately 48% increased risk of in-hospital mortality and up to a 2-fold higher risk of long-term mortality. The study demonstrated that low albumin levels were associated with an increased risk of long-term mortality [72]. Furthermore, in another multicenter registry study involving 605 patients (mean age, 64.2 years) with ischemic stroke who underwent endovascular thrombectomy (EVT), the SA levels were measured within 24 h of admission. Patients were categorized into three groups according to albumin tertiles: the first tertile (< 3.73 g/dL), the second tertile (3.73–4.02 g/dL), and the third tertile (> 4.02 g/dL). Low SA levels were reported to be independently associated with poor prognosis at 90 days after acute large-vessel occlusion stroke in the anterior circulation treated with EVT. Moreover, it was observed that patients in the lowest tertile of SA had an increased risk of poor functional outcome (OR, 2.78) and mortality (OR, 2.97) compared with those in the highest tertile. In addition, patients with lower SA levels were older and had higher systolic blood pressure and a higher prevalence of AF compared with those with higher SA levels [73].

Overall, the outcomes from these studies suggest that there is a consistent inverse relationship between SA levels and the risk of stroke.

#### Acute coronary syndrome (ACS)

ACS refers to a complex, heterogeneous clinical syndrome that includes unstable angina, non–ST-elevation myocardial infarction (NSTEMI), and ST-elevation myocardial infarction (STEMI). ACS remains a leading cause of morbidity and mortality despite advances in medical care [74]. SA plays an important role in atherothrombosis, a key event in ACS [75]. Low levels of SA have been identified as a risk factor for the development of CAD [74]. A prospective observational study was conducted involving 50 patients (mean age, 61.50 ± 9.77 years) with ACS from May 2021 to December 2021. The study reported that patients with low SA levels demonstrated worse outcomes, with the mean SA level among patients with STEMI and NSTEMI in the hypoalbuminemia group being 2.88 ± 0.11 g/dL and 3.15 ± 0.26 g/dL, respectively. Among patients who died, the mean SA level in the hypoalbuminemia group was 2.87 ± 0.06 g/dL. It was reported that patients with ACS and low SA levels demonstrated a higher risk of developing STEMI [75]. Furthermore, a study by Long et al conducted in elderly patients (n = 354; mean age, ≥ 80 years) with non–ST-elevation acute coronary syndrome (NSTE-ACS) who underwent PCI demonstrated that serum albumin-to-creatinine ratio (ACR) was an independent predictor of 1-year all-cause mortality and major adverse cardiovascular and cerebrovascular events (MACCEs). Patients with lower ACR were older and had a lower SA level. Patients in the lowest ACR tertile (which corresponds to a lower albumin level relative to creatinine) had significantly higher mortality [76].

Collectively, these findings suggest that lower SA levels are associated with adverse outcomes in ACS, particularly among elderly patients, although the evidence is primarily derived from observational studies conducted in specific clinical settings and geographical populations.

#### Hospitalized elderly patients

Hypoalbuminemia is prevalent amongst hospitalized, critically ill, and elderly patients, and has been reported in > 70% of hospitalized elderly patients [77]. Low SA levels in hospitalized elderly patients with ACS are associated with advanced age, prolonged hospitalization, and adverse clinical profiles, indicating its value as an independent prognostic marker for risk stratification and targeted treatment. Furthermore, it was reported that at the time of hospitalization, the SA level tends to decrease with age, with abnormal levels observed in patients aged 50 years and progressively worsening over time. The condition of significant and mild hypoalbuminemia was more prevalent in elderly patients aged > 65 years old [33]. A retrospective cohort study involving 2,490 consecutive patients with ACS undergoing PCI (2021–2023) evaluated the association between SA levels and the length of hospital stay. Stratification by albumin quartiles (n = 350) revealed a U-shaped relationship, indicating that both low and relatively high albumin levels were associated with prolonged hospitalization, underscoring the complex clinical interpretation of albumin in hospitalized older adults. Markedly low albumin levels likely reflect malnutrition, chronic inflammation, frailty, and reduced physiological reserve, all of which are associated with increased vulnerability to complications, delayed recovery, and prolonged hospitalization. Conversely, relatively higher albumin concentrations at admission in acutely ill elderly patients may reflect hemoconcentration secondary to dehydration or intensive diuretic therapy, conditions frequently accompanied by hemodynamic instability or renal dysfunction, which may also contribute to longer hospital stays. Therefore, elevated albumin levels in this context should not be interpreted solely as indicators of favorable nutritional status. Collectively, these findings suggest that SA functions as an integrated marker of clinical status rather than a linear predictor of outcomes, underscoring the importance of contextualized interpretation when assessing prognosis in hospitalized elderly patients [78]. After adjustment for several potential confounders, the study reported that patients with SA levels < 3.99 g/dL experienced shorter lengths of hospital stay with increasing SA levels. In addition, patients with lower SA levels were older, confirming the association between lower albumin levels and advanced age in this study. The possible mechanisms may involve low albumin levels leading to a weakened antioxidant effect, resulting in increased oxidative stress, vascular endothelial cell damage, and promotion of atherosclerosis, thereby increasing the risk of CV events. Albumin helps prevent endothelial dysfunction by regulating the integrity and function of the vascular endothelium [78].

Overall, hypoalbuminemia in hospitalized elderly patients is a prevalent and significant marker of poor CV outcomes. Its presence should help in early risk stratification and targeted interventions, particularly in elderly patients. Moreover, optimizing the treatment protocol will also help reduce hospital-related costs.

#### Other CV causes

In elderly populations, age-dependent comorbidities frequently interact with and reduce the strength of traditional CV risk factors. Thus, relying solely on conventional risk models may not precisely highlight the exact mortality risk in elderly patients. This highlights the importance of incorporating other age-related CV conditions into risk assessment. Conventional risk factors alone are not sufficient to reliably predict outcomes in the elderly. To further investigate this, a retrospective study was conducted involving patients (n = 62; mean age, ≥ 85 years) with acute STEMI, and the importance of SA was evaluated. The study indicated that SA levels have an impact on the early prognosis of older patients with STEMI [79]. In a Copenhagen General Population Study (n = 100,520; mean age, 20–100 years) with a mean follow-up of 8.5 years, it was reported that for each 1 g/dL lower albumin level (hypoalbuminemia < 3.5 g/dL), the HRs were 1.17 for ischemic heart disease, 1.25 for MI, 1.37 for any stroke, and 1.46 for ischemic stroke. The group of patients with albumin < 3.5 g/dL had a median age of 65.6 years, whereas the highest albumin group (≥ 4.5 g/dL) had a median age of about 50.8 years [80]. However, only a few studies have investigated the association between hypoalbuminemia and CV events. According to the data from the Third China National Stroke Registry (CNSR-III), low baseline SA levels (3.5 g/dL) were associated with poor outcomes and mortality in patients (n = 13,618) with acute ischemic stroke or transient ischemic attack at a 3-month follow-up period. In addition, the group of patients with albumin < 3.5 g/dL had a mean age of 68.2 ± 11.3 years [48, 15].

## Use of Artificial Intelligence (AI) and Machine Learning (ML) in CVD Management

Recent studies have started to include SA into ML models as a prognostic indicator in elderly patients with CVDs. A multicenter retrospective study involving 4,647 elderly patients with both HF and hypertension indicated that albumin, along with urea, length of hospital stay, neutrophils, and high-density lipoprotein (HDL) cholesterol, was one of the top predictors of in-hospital mortality, and the Random Forest Model achieved an area under the curve (AUC) of 0.850 [81]. In another study involving elderly patients (n = 451) with CAD combined with impaired glucose tolerance or diabetes mellitus demonstrated that albumin was a protective factor in a gradient boosting machine model that predicted 1-year mortality, with an AUC of 0.836 [82]. Beyond overall discrimination performance, model interpretability analyses provide critical insight into the prognostic relevance of individual variables within ML frameworks. In the cited Random Forest and Gradient Boosting models, Shapley additive explanations (SHAP) analyses consistently identified SA as one of the most influential predictors of in-hospital and short-term mortality among elderly patients with CVD. Lower SA levels demonstrated strong negative SHAP contributions, indicating a disproportionate impact on increasing predicted risk across a wide range of patient profiles. Notably, the high SHAP ranking of SA was independent of traditional clinical variables and remained stable across different model architectures, underscoring its robust and generalizable prognostic value. These findings suggest that SA contributes to model predictions not merely as a correlate of acute illness but as an integrative biomarker reflecting the cumulative effects of malnutrition, chronic inflammation, frailty, and diminished physiological reserve, the key pathophysiological domains that are central to CV risk stratification in older adults. The prominent SHAP weight assigned to SA therefore reinforces its clinical interpretability and biological plausibility within AI-driven prognostic models. These findings from recent studies highlight that incorporating SA into AI-driven risk prediction approaches may increase model performance and help identify high-risk elderly patients with more accuracy.

## Strengths and Limitations

This review provides several important strengths and conceptual advances relative to previously published literature. Unlike previous reviews that primarily focused on mixed-age populations, specific CVD entities, or isolated clinical settings, the present review adopts an age-focused perspective centered on older adults, in whom CV outcomes are strongly influenced by geriatric syndromes, such as frailty, malnutrition, chronic inflammation, and reduced physiological reserve. By integrating geriatric biology with CV pathophysiology, this review positions SA as an integrative biomarker rather than an isolated laboratory parameter.

In addition, this review literature search synthesizes heterogeneous SA thresholds reported across studies, highlighting the absence of a universally accepted cutoff and emphasizing the requirement for context-dependent interpretation in elderly patients. The critical evaluation of nonlinear associations, including U-shaped relationships between SA levels and outcomes, such as length of hospital stay, represents a further distinction compared with previously reported narrative summaries. Finally, the inclusion of emerging evidence on AI- and ML-based risk prediction models incorporating SA underscores the evolving role of this biomarker in precision risk stratification for older patients with CVD.

This review provides a comprehensive overview of current evidence regarding the prognostic association of SA in older patients with CVDs, including HF, CAD, AF, and hypertension/stroke. By integrating molecular and physiological insights into SA with findings from clinical studies in older patients with CVDs, this review explores pathophysiology and the potential clinical relevance of SA in patient care, offering a better understanding for clinicians and researchers. Furthermore, several large cohort and meta-analysis studies have reported an association between albumin levels and clinical outcomes across various stages of disease progression. By elucidating the relationship between nutritional status and CV outcomes, this review underscores the multifactorial nature of hypoalbuminemia in older patients with CVD, incorporating age-related physiological changes, comorbidities, and nutrition, and highlights the importance of a multidisciplinary approach to patient care and management. Despite these strengths, this review has several limitations regarding variability in albumin thresholds and heterogeneity of studies included, particularly in terms of population characteristics and outcomes. Due to substantial heterogeneity in study populations, CV conditions, albumin threshold definitions, and reported outcomes, a quantitative meta-analysis was not performed in this review. There is also a paucity of clinical studies targeting SA in elderly populations with CVDs, which limits the ability to determine whether changes in SA can improve outcomes such as survival or reduced hospitalization. Moreover, studies have only focused on observational correlation rather than mechanistic approaches that could clarify the correlation between SA levels and the outcomes of CVDs. Studies are also inconsistent in defining hypoalbuminemia, with different threshold values of SA across studies, which can complicate comparisons and clinical translation. In addition, although AI offers promising applications, its integration with SA data in older patients with CVDs remains underexplored. These findings support the need for further research to establish standardized threshold values and evaluate their potential utility in risk stratification of elderly patients with CVDs.

## Future Directions

Several important gaps remain in the current landscape of SA levels in CVDs, warranting further investigation. The present data are generally observational, making it difficult to determine whether SA is the only marker of disease severity or a modifiable risk factor with therapeutic potential. Future studies should not only address whether attenuation of hypoalbuminemia can improve clinical outcomes in older patients with CVDs but also investigate the reason for decreased albumin levels in elderly populations with CVDs. Well-designed prospective clinical trials should be conducted to target SA levels in older patients to understand age-related thresholds of SA, which may help in risk stratification of the disease. In addition, advances in AI may offer a promising approach to include SA with other clinical, biochemical, and imaging markers. The introduction of multidimensional predictive models may improve risk stratification in CVDs and support optimization of treatment strategies for elderly patients. Future studies should also evaluate the impact of hypoalbuminemia on drug binding, free drug concentrations, polypharmacy, and drug-related adverse outcomes in elderly patients with CVD.

## Conclusions

SA is a well-established, cost-effective biomarker with potential prognostic value across various CVDs, particularly in older patients. Its level in the serum is derived from multifactorial changes such as inflammation, malnutrition, comorbidities, and age-related physiology, which are prevalent in elderly populations with CVDs. Hypoalbuminemia has been associated with increased risks of mortality, hospitalization, and adverse CV outcomes in conditions such as HF, CAD, and AF. Notably, SA provides prognostic information that may inform clinical decision-making, particularly in elderly populations in whom standard biomarkers may have limited predictive potential. Future research must focus on establishing a standardized threshold, exploring mechanistic approaches, and investigating whether albumin-targeted treatment can influence the treatment-disease landscape. In conclusion, SA represents not merely a marker of underlying disease burden but an integrative prognostic biomarker that reflects nutritional status, inflammation, and physiological reserve, thereby enhancing risk stratification and informing individualized treatment strategies in high-risk elderly patients with CVD.

## Data Availability

The authors declare that data supporting the findings of this study are available within the article.

## Abbreviations

ACS: acute coronary syndrome
AF: atrial fibrillation
AMI: acute myocardial infarction
AUC: area under the curve
AI: artificial intelligence
ADHF: acute decompensated heart failure
ACR: albumin-to-creatinine ratio
BNP: B-type natriuretic peptide
CRP: C-reactive protein
CAD: coronary artery disease
CVD: cardiovascular diseases
CV: cardiovascular
CNSR-III: Third China National Stroke Registry
EVT: endovascular thrombectomy
GI: gastrointestinal
HF: heart failure
HDL: high-density lipoprotein
IL-6: interleukin-6
MACCEs: major adverse cardiovascular and cerebrovascular events
ML: machine learning
MI: myocardial infarction
NIDCM: nonischemic dilated cardiomyopathy
NSTEMI: non–ST-elevation myocardial infarction
NSTE-ACS: non–ST-elevation acute coronary syndrome
OR: odds ratio
PCI: percutaneous coronary intervention
ROS: reactive oxygen species
STEMI: ST-elevation myocardial infarction
SHAP: Shapley additive explanations
TNF-α: tumor necrosis factor-α
